# Buffering of genetic dominance by allele-specific protein complex assembly

**DOI:** 10.1126/sciadv.adf9845

**Published:** 2023-05-31

**Authors:** Mihaly Badonyi, Joseph A Marsh

**Affiliations:** MRC Human Genetics Unit, Institute of Genetics and Cancer, University of Edinburgh, Edinburgh, UK.

## Abstract

Protein complex assembly often occurs while subunits are being translated, resulting in complexes whose subunits were translated from the same mRNA in an allele-specific manner. It has thus been hypothesized that such cotranslational assembly may counter the assembly-mediated dominant-negative effect, whereby co-assembly of mutant and wild-type subunits “poisons” complex activity. Here, we show that cotranslationally assembling subunits are much less likely to be associated with autosomal dominant relative to recessive disorders, and that subunits with dominant-negative disease mutations are significantly depleted in cotranslational assembly compared to those associated with loss-of-function mutations. We also find that complexes with known dominant-negative effects tend to expose their interfaces late during translation, lessening the likelihood of cotranslational assembly. Finally, by combining complex properties with other features, we trained a computational model for predicting proteins likely to be associated with non–loss-of-function disease mechanisms, which we believe will be of considerable utility for protein variant interpretation.

## Teaser

Cotranslational assembly can counteract the dominant-negative effect by preventing the intermixing of protein complex subunits.

## INTRODUCTION

Almost half of the proteins with experimentally determined structures interact with other copies of themselves to form homomeric complexes (*1*), and more than one-third of heteromeric complexes with known structures contain sequence-identical repeated subunits (*2*). Considering the human proteome, about one-fifth of proteins have been detected to cotranslationally assemble in a simultaneous fashion (*3*), whereby two subunits interact while still being translated on the ribosome. Cotranslationally assembling homomers are thought to predominantly undergo cis-assembly, yielding allele-specific complexes (*3*–*5*). Repeated subunits in a heteromeric complex are also more likely to have come from the same transcript, especially when their assembly is seeded cotranslationally, reducing the chance of a single complex containing subunits from two alleles. We are beginning to understand the structural properties that make subunits more likely to undergo assembly on the ribosome. These include N-terminally exposed interface residues, a large interface area, a high α-helix content, and the presence of coiled-coil motifs (*3*) or domain invasion motifs (*6*). While many studies have shed light on the functional and evolutionary aspects of cotranslational assembly, our understanding of its allele-specific nature and its impact on genetics is very limited.

**Fig. 1.**
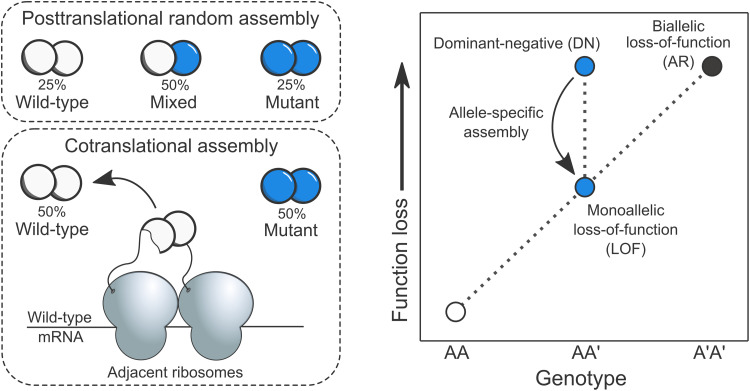
Genetic consequence of allele-specific protein complex assembly. Left: Consider a homodimer with one allele of its gene carrying a heterozygous mutation with DN properties. When complex assembly occurs after the subunits have been fully translated and folded (posttranslational random assembly), the maximum entropy configuration of subunits dictates that mixed complexes will make up half of all complexes. This means that pure wild-type and mutant complexes form only 25% of the time. However, when the homodimer cotranslationally assembles, both complexes will form independently of one another in an allele-specific manner, increasing the ratio of fully functional complexes to virtually 50%. Right: This relationship is illustrated on a phenotype versus function loss landscape diagram. Allele-specific assembly of homomers and repeated subunit heteromers may alleviate the effects of heterozygous LOF mutations by reducing the mixing between the products of wild-type and mutant genes.

Previously, a potential genetic consequence was proposed on theoretical grounds (*5*, *7*, *8*). According to the hypothesis, cotranslational assembly should reduce the likelihood of dominant-negative (DN) disease mechanisms. A DN effect occurs when expression of a mutant allele disrupts the activity of the wild-type allele (*9*, *10*), causing disproportionate function loss and thus a dominant mode of inheritance. Observational evidence has long suggested that DN effects are common in homomers (*11*), likely because incorporation of a mutant subunit into a complex along with wild-type subunits is enough to “poison” it. This assembly-mediated DN effect can lead to a reduction in functional activity exceeding the 50% that would be expected for a simple heterozygous loss-of-function (LOF) mutation. However, cotranslational assembly can result in complexes whose subunits are allele specific, i.e., made up entirely of either wild-type or mutant subunits, potentially reducing the harmful effects of an otherwise DN mutations (Fig. 1). This ability of cotranslational assembly can be considered its “buffering capacity” against DN mutations.

Some gain-of-function (GOF) mutations have a molecular mechanism similar to the assembly-mediated DN effect. At the protein-level, the phenotypic effect of GOF mutations is the consequence of the mutant protein functioning differently from the wild type, e.g., through increased protein activity. However, formation of mixed wild-type:mutant complexes can lead to GOF in a similar manner to the DN effect, but instead of the mutant blocking the activity of the wild type, the GOF is conferred to the whole complex. An example is the L171R mutation in the G protein (heterotrimeric GTP-binding protein)–activated inward rectifier potassium channel 2, implicated in the Keppen-Lubinsky syndrome, which reduces ion selectivity, thus allowing sodium and calcium to pass the channel (*12*). This mechanism can be referred to the assembly-mediated dominant-positive effect (*13*) and, just like the DN effect, should be subject to genetic buffering via allele-specific assembly. However, there are far fewer reports of this phenomenon, so most of this study will be focused on DN effects.

The detectability of genetic buffering against assembly-mediated DN and dominant-positive effects may be influenced by two molecular phenomena. First, peri-translational or localized assembly, which occurs when subunits assemble shortly after translation near the parent mRNA (*7*), could also make it more likely that subunits of a complex are allele specific. This effect is likely to be more common in highly abundant proteins, whose transcripts have high ribosome densities and are translated more efficiently than those of lowly abundant proteins (*14*, *15*). Second, subunit exchange may increase the entropy of subunit stoichiometry post-assembly via shuffling of wild-type and mutant subunits (*16*), resulting in proportions expected from random posttranslational assembly. However, because the likelihood of subunit exchange is determined by the dissociation constants of the subunits involved, it should be less likely to occur in cotranslationally assembling complexes, which tend to have larger interfaces (*17*) and thus generally higher binding affinities.

Overall, cotranslational assembly may counter the DN effect, which can have an appreciable impact on how genes with inherited and de novo missense variants are prioritized in clinical sequencing pipelines. To address this idea, we used a set of experimentally determined cotranslationally assembling proteins and formulated two hypotheses based on the above lines of thought. First, genes with an autosomal dominant (AD) disease inheritance pattern should be less likely to assemble cotranslationally compared to autosomal recessive (AR) genes, given that a large fraction of them are likely to be associated with DN effects. Second, protein subunits with known DN disease mutations should have the lowest rate of cotranslational assembly compared to other genes with AD inheritance. Here, we show that both hypotheses are upheld. Examination of the structural properties of complexes associated with DN mutations suggests that their interfaces are exposed relatively late during translation, which should strongly disfavor cotranslational assembly. Using a knowledge-based approach, we trained a regression model to prioritize genes whose mutations are expected to be associated with non-LOF disease mechanisms. We hope that our work will be of interest to clinical geneticists and accelerate the prediction and discovery of variant-level molecular mechanisms.

## RESULTS

### AD genes are depleted in cotranslationally assembling subunits

We started with a set of 9053 human proteins, of which 6562 (72%) physically interact with copies of themselves to form homomeric complexes. The remaining 2491 (28%) are repeated subunits of heteromers, meaning that they are present in heteromeric complexes in more than one copy. Both types of proteins have the potential to be associated with assembly-mediated DN or dominant-positive effects, as the mutant and wild-type proteins can co-assemble within the same complex. We obtained genetic inheritance modes from the OMIM database (*18*) and defined a gene as AD if it had any disease inherited in an AD pattern, which could possibly be caused by assembly-mediated DN or dominant-positive mutations. We defined a gene as AR if it had mutations inherited exclusively in an AR pattern, which are almost certain to be associated only with LOF.

**Fig. 2.**
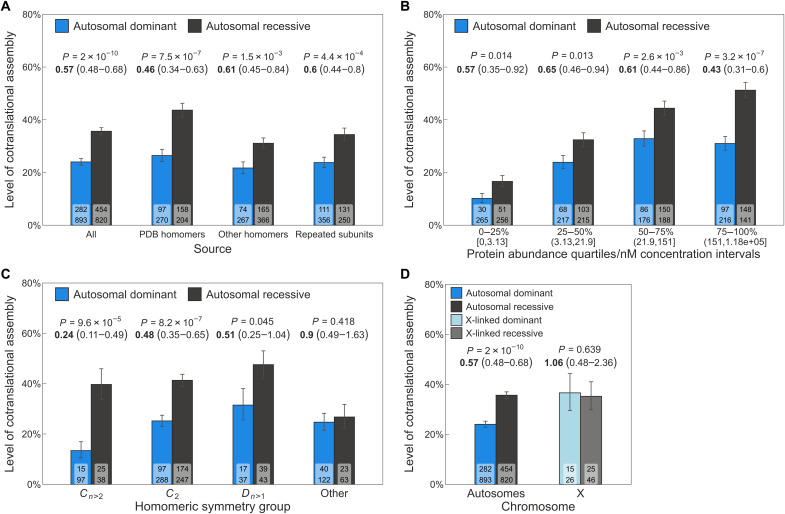
AD genes are depleted in cotranslationally assembling subunits. (**A**) Level of cotranslational assembly in homomers and repeated subunits among AD versus AR genes grouped by subunit source (see Methods). Bar values are percent level of cotranslational assembly; error bars are Jeffrey’s 68% binomial credible intervals. The *P* value from the hypergeometric test and the OR (in bold) and its 95% confidence interval are shown above the bars. Labels on bars are the count of cotranslationally assembling subunits (top) and all other subunits (bottom). (B) to (D) have the same parameters. (**B**) Level of cotranslational assembly binned into protein abundance quartiles. Each bin corresponds to 25% of proteins by count, and the corresponding approximate nanomolar concentration intervals are shown in brackets. (**C**) Level of cotranslational assembly in genes of homomers and repeated subunits with AD and AR disease inheritance split by symmetry groups: cyclic (*C*_*n*>2_), twofold (*C*_2_), dihedral (*D*_*n*>1_), and other. (**D**) Comparison of the level of cotranslational assembly in genes of homomers and repeated subunits on autosomes or the X chromosome.

Our initial hypothesis was that, among human disease-associated genes that encode homomers or repeated subunits of heteromers, those known to exhibit AD inheritance would have lower levels of cotranslational assembly compared to those with exclusively AR inheritance. Although using AD inheritance as a proxy for the DN effect is a simplification, assembly-mediated DN effects are believed to play an important role in AD disorders (*19*). Our analysis shows that 24% of AD subunits undergo cotranslational assembly compared to 35.6% with AR inheritance (*P* = 2 × 10^−10^, hypergeometric test). We calculated the odds ratio (OR) to assess the strength of the difference between the groups. Overall, the OR of 0.57 implies that the odds of cotranslational assembly for a randomly selected AD subunit are almost half as that for an AR subunit. In Fig. 2A, we show this analysis grouped by the three main sources of the subunits: homomers with experimentally characterized structures [Protein Data Bank (PDB) homomers], homomers with nonstructural evidence (other, which includes SWISS-MODEL homology models and evidence for homo-oligomerization from different databases), and repeated subunits of heteromers (see Methods). The strongest effect was found in PDB homomers (OR = 0.46, *P* = 7.5 × 10^−7^), followed by repeated subunits (OR = 0.6, *P* = 4.4 × 10^−4^) and other homomers (OR = 0.61, *P* = 1.5 × 10^−3^). We speculate that the stronger trend in PDB homomers is due to their enrichment in biologically important interfaces, making a higher fraction of this group compatible with the buffering of assembly-mediated effects. Nonetheless, the results show that the trend is consistent across all groups, despite slight variations in effect size.

Protein abundance can bias both cotranslational assembly and its detection. Ribosome density tends to be higher in abundant proteins (*14*), resulting in more ribosome footprints for sequencing. Proteins that are more abundant might exhibit a higher level of peri-translational assembly, which could also affect the degree of allele-specific complex formation. Therefore, it is important to control for abundance in our analysis, in case the enrichment of highly abundant proteins in the cotranslationally assembling group is affecting our results. We examined the median abundance of AD and AR homomers and repeated subunits, finding no significant difference between them (fig. S1A; *P* = 0.658, Wilcoxon rank sum test). We then divided subunits into quartiles based on their approximate intracellular concentration, ranging from 0.005 nM to 180 μM, and found that the trend of AD subunits having lower cotranslational assembly rates than AR subunits held across all quartiles, with the strongest effect in the highest abundance bin (OR = 0.43, *P* = 3.2 × 10^−7^) (Fig. 2B). These results were mirrored by a complementary analysis using active ribosome-protected fragment counts specific to human embryonic kidney (HEK) 293 cells (fig. S1B) (*20*), used by (*3*) for the detection of cotranslational assembling proteins.

Symmetry plays an important role in the formation of homomers, with each symmetry group exhibiting unique sequence and structural features that influence their functional roles (*1*, *21*). The three most common symmetry groups in the human proteome are twofold (Schönflies notation: *C*_2_), higher-order cyclic (*C*_*n*>2_), and dihedral symmetry (*D*_*n*>1_). In a previous study, we showed that cotranslationally forming complexes tend to have large interfaces across the symmetry groups (*17*). However, there are also symmetry-level differences, with members of the cyclic symmetry being the least likely to undergo cotranslational assembly (fig. S1C). When we divided the AD and AR homomers based on their symmetry group, we observed substantial variation in the level of cotranslational assembly (Fig. 2C). For instance, if we randomly select a cyclic complex with AD inheritance, the odds of cotranslational assembly are 0.24 times lower compared to an AR cyclic complex (*P* = 9.6 × 10^−5^). In contrast, the odds of cotranslational assembly for a dihedral complex are only 0.51 times lower (*P* = 0.045). Symmetries that have a low representation in the human proteome, such as helical, cubic, and asymmetric homomers, were grouped into the “other” category. We did not find a significant trend in this group, which may be due to the heterogeneous properties of their members.

We also considered the genetic dominance of mutations on autosomes and the X chromosome. Autosomal genes typically exist in two copies, either homozygous or heterozygous, with one allele on each chromosome. Genes on the X chromosome, on the other hand, are hemizygous in males and present in two copies in females, where one allele is usually silenced, excluding a subset of genes that escape X-inactivation in a tissue-specific manner. This means that cotranslational assembly is unlikely to be effective in buffering X-linked dominance, since there is no wild-type allele to counteract the phenotype. Our findings support this idea, as we did not find a significant difference in the level of cotranslational assembly between X-linked dominant and recessive genes of homomers and repeated subunits (Fig. 2D; OR = 1.06, *P* = 0.639).

Finally, we examined a range of confounding variables to ensure the robustness of the results, including protein length, presence of coiled-coil motifs, and the confidence-based classification of cotranslationally assembling proteins (*3*). Our analyses suggest that none of these factors have a significant impact on the trend (Supplementary Text), reinforcing the idea that the allele-specific assembly of protein complex constituents can act as a buffer for certain dominant mutations.

### Subunits with DN disease mutations are less likely to assemble cotranslationally than subunits with heterozygous LOF mutations

Given the lower occurrence of cotranslational assembly in AD genes compared to AR genes, we sought to investigate further the molecular mechanisms underlying this trend. Nearly all mutations with dominant inheritance cause disease via one of three broad mechanisms: heterozygous LOF (or haploinsufficiency), GOF, and DN (*13*). We hypothesized that subunits with DN disease mutations should show a reduction in cotranslational assembly compared to genes with LOF mutations. This is because cotranslational assembly reduces the mixing of wild-type and mutant subunits, therefore lessening the likelihood that the mutant will interfere with the function of the wild type and inflict a DN effect.

**Fig. 3.**
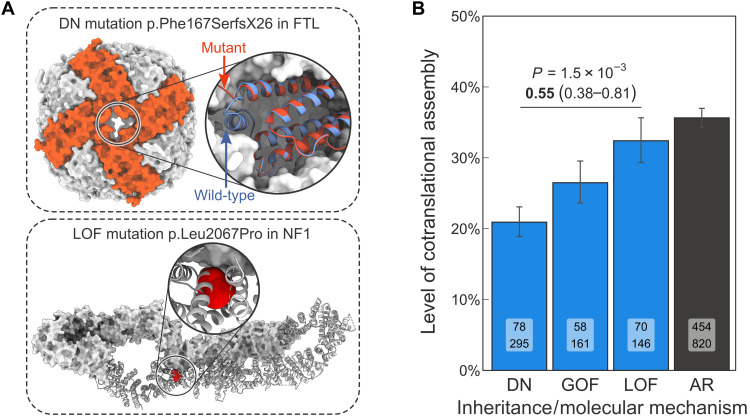
Subunits with DN disease mutations are less likely to assemble cotranslationally than subunits with heterozygous LOF mutations. (**A**) Known examples of protein-level genetic disease mechanisms. Top: Structure of the p.Phe167SerfX26 mutant ferritin light chain complex (PDB ID: 4v6b) overlaid on the wild type (2ffx). Bottom: Structure of the wild-type neurofibromin 1 dimer (7mp6) shown as surface (top subunit) and as cartoon (bottom subunit), with the LOF mutation–associated residue Leu^2067^ highlighted. (**B**) Level of cotranslational assembly in homomers and repeated subunit heteromers according to dominant molecular mechanisms and AR inheritance. Bar values are percent level of cotranslational assembly; error bars are Jeffrey’s 68% binomial credible intervals. The *P* value from the hypergeometric test and the OR (in bold) and its 95% confidence interval are shown for the DN versus LOF comparison. Labels on bars are the count of cotranslationally assembling subunits (top) and all other subunits (bottom).

To begin, we classified 1185 AD genes (66% of known AD genes, listed in table S1) into LOF, GOF, and DN mechanisms using text-mining approaches and manual curation of the corresponding evidence (detailed in Methods). For example, a DN mutation in the ferritin light chain complex, which stores iron in a readily available form, has been linked to neurodegenerative disorders associated with iron accumulation in the brain (*22*). Specifically, the F167SfsX26 mutation replaces a C-terminal short helix with a stretch of disordered residues, which is thought to have a DN effect by creating large pores in the complex (Fig. 3A), thus affecting its iron storage ability. Although the mutation has severe functional consequences, it does not impede assembly. By contrast, the LOF mutation L2067P in neurofibromin 1, observed in spinal neurofibromatosis (*23*), affects the protein’s folded core and dimer interface (Fig. 3A). Mutations like these may escape nonsense-mediated decay and either lead to aggregation of the protein before assembly can occur or render its interface incompatible with assembly, hence have no effect on the wild type.

We first evaluated the suitability of the gene sets for analysis by examining properties known to be associated with the different molecular mechanisms, such as the change in Gibbs free energy (ΔΔ*G*) upon pathogenic mutations and their clustering in three-dimensional (3D) space (*24*). It was previously observed in a subset of membrane proteins that DN mutations tend to have low predicted ΔΔ*G* values, consistent with the fact that the mutant protein needs to remain stable enough to assemble into complexes (*25*). In agreement with this, we found that DN mutations in homomers and repeated subunits have significantly lower predicted ΔΔ*G* values compared to LOF mutations (fig. S2A; *P* = 1.3 × 10^−16^, Wilcoxon rank sum test). In terms of 3D clustering, non-LOF mutations are often concentrated in specific regions of a protein, such as interfaces and functional sites, while LOF mutations tend to be more dispersed throughout the structure (*24*). Consistent with this, DN mutations in our data exhibit higher 3D clustering than LOF mutations (fig. S2B; *P* = 4.7 × 10^−4^, Wilcoxon rank sum test) and are enriched at homomeric interfaces (fig. S2C; *P* = 1.3 × 10^−18^, hypergeometric test).

We next directly addressed our hypothesis by calculating the fraction of cotranslationally assembling subunits in each molecular mechanism group, shown in Fig. 3B. As expected, the fraction is markedly lower among DN (20.9%) compared to LOF subunits (32.4%; OR = 0.55; *P* = 1.5 × 10^−3^, hypergeometric test) and AR subunits (35.6%, OR = 0.49, *P* = 6.9 × 10^−8^). At the molecular level, AR and heterozygous LOF mutations are very similar in their effect. Recessive disorders are almost always due to biallelic (homozygous or compound heterozygous) LOF, with a few rare examples of biallelic GOF (*26*–*28*). Our results indicate that the level of cotranslational assembly in subunits with monoallelic and biallelic LOF mutations is similar, but subunits with DN mutations are observed to assemble cotranslationally less frequently. Therefore, allele-specific protein complex assembly may prevent some mutations from the clinical manifestation of a phenotype caused by certain heterozygous variants. While there is no statistically significant difference in cotranslational assembly between the GOF and LOF classes (26.5% versus 32.4%, *P* = 0.11), we observed a significant depletion in the GOF class relative to AR (26.5% versus 35.6%, OR = 0.65, *P* = 4.7 × 10^−3^). We speculate that this discrepancy may be due to the assembly-mediated dominant-positive effect that often underlies GOF mutations (*13*).

Cyclic symmetry is found at a much higher frequency in GOF homomers than in the LOF class (fig. S2D; 21.3% versus 6.4%; *P* = 3.9 × 10^−3^, Fisher’s exact test). This symmetry group has the lowest level of cotranslational assembly (18.4%) in comparison to twofold homodimers (27.3%) and dihedral complexes (31.2%) (fig. S1C). For this reason, we investigated the potential confounding effect of structural symmetry and found distinct preferences among the molecular mechanisms (fig. S2D). DN homomers, similar to GOF, are enriched in cyclic symmetry relative to LOF (20.4% versus 6.4%; *P* = 4 × 10^−3^, Fisher’s exact test). However, dihedral symmetry is markedly enriched in AR homomers compared to DN homomers (12.6% versus 4.8%; *P* = 1.3 × 10^−3^, Fisher’s exact test). We suspect that these symmetry group compositions are reflective of biases in protein function, because the relationship between disease mechanisms and protein function is well established. For example, disorders caused by genes encoding enzymes are primarily recessive (*29*), genes encoding transcription factors are more likely to be haploinsufficient (*30*), and those of membrane channels commonly give rise to non-LOF disease mechanisms (*31*). These admittedly broad functional classes have been linked to structural properties, with metabolic enzymes being enriched in dihedral symmetry, transcription factors in twofold symmetry, and membrane channels in cyclic symmetry (*1*, *21*, *32*). Our investigation into protein functional classification confirmed these assumptions (fig. S2E). Metabolic enzymes are overrepresented in AR subunits (AR versus all other; OR = 4.27; *P* = 3.9 × 10^−40^, Holm-Bonferroni corrected Fisher’s exact test) and membrane transporters among GOF and DN subunits (OR = 2.96, *P* = 4.8 × 10^−9^ and OR = 1.99, *P* = 4.8 × 10^−6^, respectively), and transcription factors have 6.9-fold higher odds to be associated with the LOF class than a subunit sampled randomly from the disease gene pool (*P* = 2.15 × 10^−19^). When homomers in the different molecular mechanism classes are grouped by their symmetry, the level of cotranslational assembly is consistently lower among DN subunits than in LOF or AR subunits (fig. S2F). The only exception is the LOF class with cyclic symmetry, where the rate of cotranslational assembly cannot be reliably estimated because no cotranslationally assembling member has been identified.

Last, we split the analysis into homomers and repeated subunits of heteromers. We found that repeated subunits with DN mutations exhibit a nonsignificant reduction in cotranslational assembly relative to the LOF class (fig. S2G; OR = 0.74, *P* = 0.188) and a significant reduction relative to the AR class (OR = 0.55, *P* = 3.3 × 10^−3^). As expected, homomers with DN disease mutations are more strongly depleted in cotranslational assembly compared to these classes (OR = 0.44, *P* = 1.1 × 10^−3^ and OR = 0.43, *P* = 2 × 10^−6^, respectively). Overall, the results demonstrate that the genetic buffering capacity of cotranslational assembly, despite evident differences in structural symmetry, is neither confounded by symmetry nor exclusive to homomers, but extends to repeated subunits of heteromeric complexes.

### Interfaces of homodimers with DN disease mutations are C-terminally shifted

To further understand the observation that subunits with DN disease mutations are less likely to undergo cotranslational assembly, we investigated the impact of interface area, which we have previously established as an important correlate of cotranslational assembly (*17*). Homomeric complexes with larger subunit contact areas are more hydrophobic and experience a stronger drive to assemble early on the ribosome. Because of the known confounds of structural symmetry, we performed the analysis split by homomeric symmetry groups. The analysis revealed that subunits associated with DN mutations do not have smaller interfaces than other disease-related subunits (fig. S3A). On the contrary, the interfaces of LOF homomers are significantly smaller relative to DN subunits across the main symmetry groups. This finding is consistent with the enrichment of pathogenic mutations at interfaces of DN subunits (fig. S2C), which, assuming a random mutation model, would be less likely to occur if the interfaces were small. However, larger interface areas for GOF, DN, and AD/AR subunits could also reflect the biological importance of their interfaces, given that their molecular mechanisms depend on complex formation. Conversely, subunits in the LOF group may have a higher proportion of crystallographic interfaces, which are typically smaller (*33*).

**Fig. 4.**
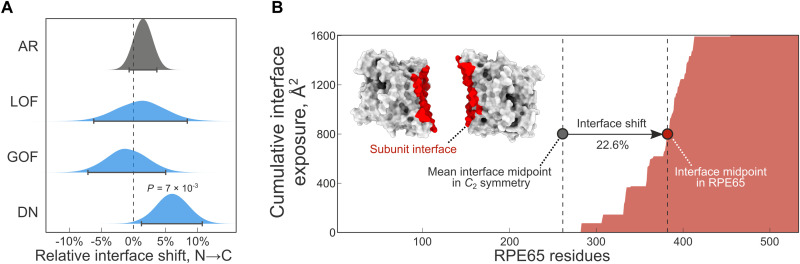
Interfaces of homodimers with DN disease mutations are C-terminally shifted. (**A**) Bootstrap distributions of the difference between the relative interface location of the symmetry mean and the observed value for *C*_2_ symmetric homodimers in the different classes. Error bars represent 95% confidence intervals of the percentile method, and the *P* value was calculated from the resamples. (**B**) Cumulative interface exposure of the enzyme RPE65 during the translation process. Half of its final interface area (1604 Å^2^) is reached 22.6% later than what is expected from in the *C*_2_ symmetry group.

We next examined the interface location of the subunits, because the idea that N-terminal regions of proteins are more likely to be involved in cotranslational interactions has received strong experimental support (*3*, *34*–*36*). We hypothesized that interfaces of homomeric subunits with DN disease mutations should be C-terminally shifted, reflective of their lower tendency to assemble cotranslationally. To test this, we calculated the relative interface location for all homomeric subunits and the average interface location for the different symmetry groups (fig. S3B) (*17*). We found that the interfaces of homodimers with DN disease mutations are significantly more C-terminal compared to what is expected from the symmetry group (*P* = 0.025, Holm-Bonferroni corrected Wilcoxon rank sum test against basemean). To quantify this difference, we resampled the homodimer dataset with replacement, stratified by molecular mechanisms, and calculated confidence intervals. Figure 4A shows the bootstrap distribution of the relative interface shift, i.e., distance from the symmetry mean (raw relative interface location values are shown in fig. S3C). The relative interface shift can be interpreted as a percentage, where +5% indicates that the interface is C-terminal to its expected value by 5% of the protein’s length. According to this, subunits of homodimers with DN disease mutations are 6% more C-terminal compared to the symmetry group mean (*P* = 7 × 10^−3^, resampling *P* value).

We exemplify this finding in Fig. 4B. The retinoid isomerohydrolase (RPE65) is an enzyme critical for phototransduction in the retinal pigment epithelium. Retinitis pigmentosa has been linked to both recessive and dominant mutations in RPE65, making heterozygous mutations in the gene less likely to be caused by simple LOF mechanisms. The DN mutation D477G was found to exert a DN effect and delay chromophore regeneration (*37*). Notably, the interface of RPE65 during translation is exposed 22.6% later than what would be expected from an average homodimer, which creates a condition that disfavors cotranslational assembly. The observation is consistent with the absence of cotranslational assembly in RPE65 (*3*) and supports a model whereby subunits that expose their interfaces later in the translation process are less likely to assemble cotranslationally and are, in turn, more susceptible to DN mutations.

### Genes associated with non-loss-of-function molecular disease mechanisms can be computationally predicted

Many studies have aimed at understanding the properties of haploinsufficient genes (*38*–*43*). However, comparatively little effort has been channeled into exploring the characteristics of genes that give rise to dominant disorders in a manner not explained by simple LOF. Recently, we have shown that state-of-the-art variant effect predictors show worse performance in genes associated with non-LOF mechanisms (*24*). This observation emphasizes the importance of considering alternative molecular mechanisms in our collective attempt to annotate human pathogenic variation. While the text-mining strategy we used here was able to provide likely molecular mechanism assignments for 1185 dominant disease genes, many others remain unknown. Moreover, for as-of-yet undiscovered disease genes, there is a strong possibility that we could miss pathogenic mutations associated with DN or GOF mechanisms due to the difficulties in computationally predicting their effects. To facilitate future variant-level molecular mechanism prediction and aid clinical geneticists in evaluating the potential of mutations to inflict a non-LOF consequence on the protein, we built a classifier with the goal of prioritizing genes most likely to be associated with non-LOF mechanisms over those primarily associated with LOF.

**Fig. 5.**
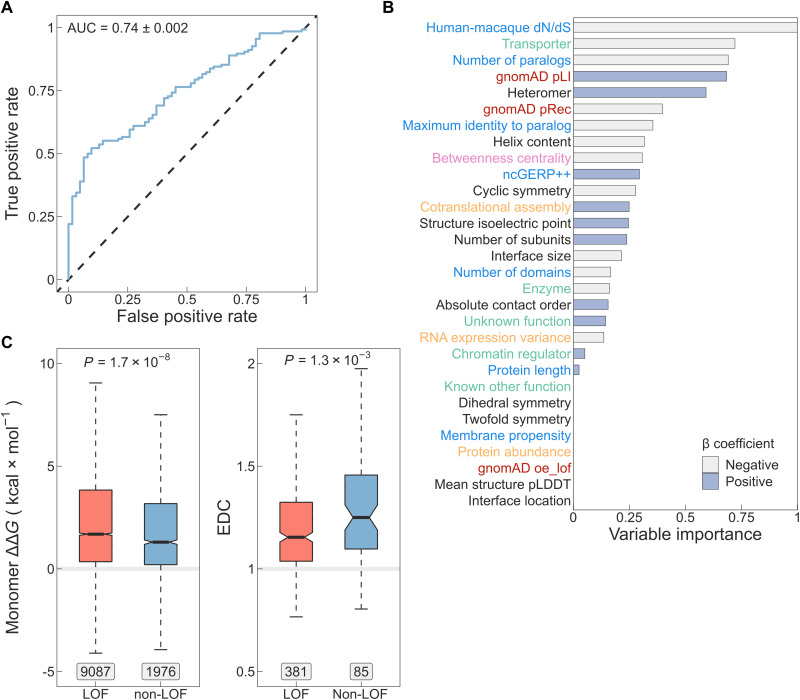
A computational model for identifying genes most likely to be associated with non-LOF molecular mechanisms. (**A**) Receiver operating characteristic (ROC) curve of the lasso regression model measured on the test set. AUC ± bootstrap (*n* = 1000) SE is shown. (**B**) Variable importance calculated as the absolute values of the β coefficients scaled to the [0,1] interval. The *y*-axis labels are colored according to the type of the variable: sequence-derived or evolutionary variables (blue), functional annotations (green), mutational constraint metrics (red), structural properties (black), interaction network–based property (pink), and experimental data (orange). Bars are colored based on the sign of β. (**C**) Differences in Gibbs free energy change (ΔΔ*G*, left) and EDC (right) of pathogenic mutations between genes predicted to be non-LOF versus all other genes at threshold T2. Genes that were used for training the model as well as known AR genes were excluded. Boxes denote data within 25th and 75th percentiles, the middle line represents the median, the notch contains the 95% confidence interval of the median, and the whiskers extend from the upper and lower quartiles to a distance of 1.5 times the interquartile range. Labels indicate the number of variants (for ΔΔ*G*) or the number of genes (for EDC) in the groups. The *P* values were calculated with the Wilcoxon rank sum test.

We first reviewed the literature to identify properties of LOF genes and then trained a logistic regression model with lasso penalty using a range of diverse features. These features encompassed cotranslational assembly (*3*), the functional and structural characteristics evaluated in this study, population-level mutational constraints (*40*), as well as evolutionary, sequence-based, interaction network–based attributes (*39*, *42*, *43*) and experimental data (*44*, *45*) (detailed in Methods). Measured on the test set, the classifier achieves an area under the receiver operating characteristic (ROC) curve of 0.74 (Fig. 5A), an *F*_1_ score of 0.8, and a Matthews correlation coefficient of 0.24 (detailed performance profile in fig. S4). Cotranslational assembly was found to be a discriminating feature in the model, ranking 12th out of 30 features and being roughly one-fourth as important as the top predictor, which is the ratio of nonsynonymous-to-synonymous substitutions (dN/dS) in the coding sequence of human relative to macaque genes (*39*) (Fig. 5B). Notably, the second and third most important predictors in the model are transporter/channel function and the number of paralogs of the gene. It has been observed before that haplosufficient genes have higher average sequence identity to the closest paralog than LOF genes (*39*), suggesting functional compensation by closely related proteins. It is possible that due to this functional redundancy, AD genes with many paralogs are simply more likely to be associated with non-LOF mechanisms. For example, ion channel genes are known to have undergone multiple gene duplication events (*46*), which is consistent with their enrichment among DN and GOF subunits (fig. S2, D and E). In cyclic complexes with more than one unique subunit, paralogous copies typically sequester in the same complex (*47*), suggesting that information on paralogs is a valuable proxy for non-LOF mechanisms in homomers as well as in heteromers.

We derived two probability thresholds for the predictions (fig. S4E). The threshold of *P* = 0.82 (T1) was selected on the basis of the maximum value of Youden’s J statistic (*48*) [test set confusion matrix: 68/68 (50%) non-LOF versus 5/57 (8%) LOF]. A second threshold of *P* = 0.92 (T2) was chosen as the value at which the specificity of the model reaches 100%, i.e., no ground-truth LOF genes are classified as non-LOF at the cost of classifying more ground-truth non-LOF genes as LOF [29/107 (21%) non-LOF versus 0/62 (0%) LOF]. We provide predictions for 9051 proteins covering ~44% of the proteome (table S2) that have at least partial structures in the PDB. Of these, 880 (9.7%) are above T2 and 3315 (36.6%) are above T1. Of the latter, 2840 have no dominant disease association recorded in OMIM.

As an unbiased approach to assess the model, we analyzed the ΔΔ*G* of pathogenic mutations and their extent of disease clustering (EDC) after removing genes used for training, and AR genes, whose biallelic LOF mutations would bias the trend. In Fig. 5C, we show the result of this analysis at threshold T2, demonstrating that missense mutations in predicted non-LOF genes exhibit a milder impact on protein structure (*24*, *25*). Moreover, pathogenic variants in predicted non-LOF genes show strong 3D clustering in their respective protein structures, consistent with previous observations (*24*). In fig. S4 (F and G), we provide further support that, between thresholds T1 and T2, both ΔΔ*G* and EDC exhibit the expected trend with an increasing effect size.

## DISCUSSION

Cotranslational assembly of homomers is thought to result in complexes whose subunits originate from the same allele (*3*, *4*, *49*). A possible consequence of this mechanism is that subunits harboring pathogenic heterozygous mutations may be sequestered into half of the protein complex pool rather than mixing with the wild type and inflicting functionally harmful effects. By comparing the fraction of cotranslationally assembling subunits associated with Mendelian diseases, we showed that genes of homomers and repeated subunits with mutations inherited in an AD pattern are significantly depleted in this mode of assembly compared to AR genes. Moreover, among AD genes of homomers, those that exert a DN effect are the least likely to cotranslationally assemble compared to other protein-level molecular mechanisms of disease, but importantly, to those that predominantly harbor heterozygous LOF mutations. Our results therefore reveal a previously hypothesized genetic buffering mechanism (*7*, *8*), whereby complexes undergoing cotranslational assembly are to some extent protected from the deleterious consequences of DN mutations.

We observe AR complexes to have consistently high levels of cotranslational assembly regardless of their structural symmetry. It was first proposed by Wright (*50*) and Haldane (*51*), whose ideas were developed further by Hurst and Randerson (*52*), that recessivity is a consequence of selection for larger amounts of protein, because the high abundance of enzymes is a “safety factor” (*53*) that increases their robustness to dominant mutations. It is possible that the abundance and the structural properties of metabolic enzymes, such as their preference for dihedral symmetry (*1*), necessarily lead to frequent cotranslational assembly events, representing an additional safety factor against the deleteriousness of dominant, especially DN, mutations. Although the evolution of protein oligomeric state can arise from nonadaptive processes (*54*, *55*), it is not implausible that biological phenomena such as this impose weak selection.

Our results hint at the extraordinary regulation of protein complex assembly within cells. Allele-specific assembly in homomers may emerge from the inherent colocalization of their nascent chains, although certain protein structural features appear to predispose subunits to the process. We also observed repeated subunits of heteromeric complexes to exhibit genetic buffering by cotranslational assembly. According to one hypothesis, subunits may combine information in their mRNAs and protein sequences to increase the efficiency of assembly mediated by RNA binding proteins (*56*–*58*). A range of membraneless compartments have been put forward as putative sites of intense protein complex assembly under physiological conditions, including TIS granules (*59*), assemblysomes (*60*), and translation factories (*61*), which may well represent the same type of organelles [reviewed in (*62*)].

**Fig. 6.**
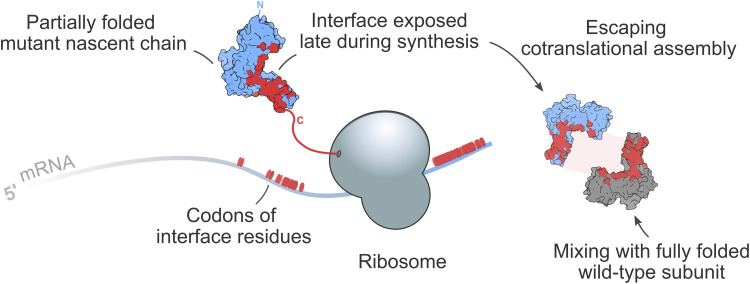
Mechanistic interpretation of C-terminally shifted interfaces in homodimers with DN mutations. Schematic representation of a structural trend underlying pathogenic DN effects. A mutant subunit is more likely to assemble posttranslationally when it exposes its interface residues late in the translation process, which can increase the level of mixing with the wild-type subunit.

Across diverse proteomes, interface contacts of homomers are enriched toward the C terminus, which is thought to be the product of evolutionary pressure on folding to happen before assembly (*35*). By contrast, N-terminal protein interfaces have been found to favor cotranslational assembly (*3*, *17*, *34*–*36*). Our structural analysis suggests that interfaces of homodimers with DN disease mutations are significantly shifted toward the C terminus relative to what is expected from their symmetry group. As a possible consequence, their interfaces become exposed in nascent polypeptides relatively late during translation, strongly reducing the likelihood of cotranslational assembly, as illustrated in Fig. 6. This observation represents a survivorship bias so that we tend to observe that subunits cause disease via a DN mechanism when they “escape” cotranslational assembly and subsequently co-assemble with wild-type subunits.

Ongoing efforts to develop variant effect predictors focusing on the molecular consequences of protein-coding variants should consider whether the subunit assembles cotranslationally or, providing that structural data are available, the properties of interfaces to prioritize those with a possible DN effect. As demonstrated in this study, a substantial fraction of subunits with mutations inherited in an AD pattern display a DN phenotype, likely including many of those that have not yet been characterized and classified under one of the molecular mechanism classes. Additionally, current clinical sequencing pipelines frequently identify inherited and de novo heterozygous variants in recessive genes, which are ranked lower under the assumption that they would not be pathogenic in a heterozygous state (*63*–*68*). However, a DN effect is possible if the gene encodes a homomer or repeated subunit heteromer (*13*), and especially if its complex does not assemble cotranslationally. Here, covering almost half of the human proteome, we provide predictions of genes most likely to be associated with non-LOF mechanisms to expedite the discovery of variants associated with alternative molecular disease mechanisms.

Ultimately, our results shine light onto the fascinating connection between inheritance, which determines the genetic traits of an individual, and protein complex assembly, which takes place only after the genetic information has been decoded. Further research is needed to measure directly the effect of allele-specific assembly on wild-type and mutant subunits in vivo.

## METHODS

### Structural data

We searched the PDB (https://www.rcsb.org/) on 18 February 2021 for all polypeptide chains >50 amino acids and >90% sequence identity to human canonical sequences in the UniProt proteome UP000005640. For genes that map to multiple chains, we selected a single chain ranking by sequence identity, the number of unique subunits in the complex, and the number of atoms present in the chain. In every case, we used the first biological assembly and its symmetry assignment was taken from the PDB. The interface area was calculated at residue-level between all pairs of subunits with AREAIMOL from the CCP4 suite (https://www.ccp4.ac.uk/), using a probe radius of 1.4 Å. The interface was defined as the difference between the solvent-accessible surface area of the subunit in isolation and within the context of the full complex. Subunits with interfaces >400 Å^2^ were considered for analysis to exclude potentially crystallographic interfaces.

We extended the PDB dataset with homology models of human homomeric complexes in the SWISS-MODEL repository (https://swissmodel.expasy.org/repository/) (UniProt release 2022_02). Models based on isoform sequences were excluded. The software AnAnaS (https://team.inria.fr/nano-d/software/ananas/) was run on default settings to determine the number of subunits and the symmetry group of the complexes. In rare cases when symmetry was not detected, we assigned the symmetry group of the PDB template used to model the complex. If a protein was found in multiple homology models, we selected the one with the largest number of subunits followed by the length of the modeled chain. The interface area was calculated at residue level between all pairs of subunits with FreeSASA 2.0.3 (http://freesasa.github.io/) using a probe radius of 1.4 Å. We performed pairwise alignments between the modeled chain and the paired UniProt sequence to confirm residue correspondence to the canonical sequence, because any mismatch in residue numbering could influence the relative interface location metric. Similarly to the PDB structure data, only subunits with interfaces >400 Å^2^ were included in the analyses. When the SWISS-MODEL dataset was pooled with the PDB dataset, we prioritized the homomeric subunit with the larger interface area.

### Relative interface location

The relative interface location is a value between 0 and 1 indicating the location of the interface relative to the protein termini (N = 0 and C = 1), and it was calculated as previously described (*17*). To ensure that the analysis is not biased by homologous proteins, we generated a distance matrix based on the sequences of the chains from the structures using Clustal Omega version 1.2.4 (http://www.clustal.org/omega/). The distances were converted to percent identities, and the matrix was filtered to below 50% using a redundancy-filtering algorithm. Only those structures were included in the analysis that passed the homology cutoff.

### FoldX free energy calculation

FoldX 5.0 (https://foldxsuite.crg.eu/) was used to calculate the change in Gibbs free energy of ClinVar (*69*) missense mutations in AlphaFold predicted structures of human proteins (https://alphafold.ebi.ac.uk/). The “RepairPDB” command was first run to minimize structures followed by the “BuildModel” command on the repaired structures. The final Gibbs free energy change was calculated as the average of 10 replicates, and in subsequent analyses, residues with per-residue conficence scores (pLDDT) < 50, which are predicted to be disordered in solution (*70*), were excluded.

### 3D clustering of missense pathogenic mutations

The EDC metric expresses the proximity of every disease nonassociated protein residue to a known disease-associated residue, and it was calculated as previously described from AlphaFold predicted structures (*24*). Briefly, for each residue with pLDDT > 50, we calculated the α-carbon distance to all other residues with a known ClinVar disease mutation, selecting the shortest distance. The final metric is derived as the ratio of the common logarithm of nondisease and disease average distances. Values ≤1 indicate that the mutations are dispersed, and those >1 suggest a degree of spatial clustering. Only proteins with at least three pathogenic or likely pathogenic missense mutations in ClinVar were included.

### α-Helix content

The percentage of α-helix residues was calculated from the AlphaFold predicted structures of human proteins using DSSP version 2.2.1 (https://swift.cmbi.umcn.nl/gv/dssp/).

### Gene-level inheritance patterns

Gene-disease inheritance relationships were obtained from OMIM (*18*). Gene-specific XML files were retrieved via the OMIM API in four batches over consecutive days ending on 7 July 2022. Inheritances were extracted from the “phenotypeInheritance” node of each XML file.

### Gene set of homomers and repeated subunits

We extended the gene set of homomers identified by our structural mapping pipeline with genes that have nonstructural evidence to form homo-oligomers or are present in >1 copy in a complex. For homomers, we used UniProt (*71*), EMBL-EBI ComplexPortal (*72*), CORUM (*73*), the OmniPath database (*74*), as well as single spanning membrane homodimers from the Membranome 3.0 database (*75*). For repeated subunits, we extracted protein chains that appear in multiple copies in the biological units of complexes in the PDB (*2*), and included proteins that have a stoichiometry of >1 in the OmniPath database. Homomers were removed from the repeated subunit list to create a nonredundant dataset.

### Gene-level classification of dominant molecular mechanisms

We classified AD genes into molecular disease mechanisms via text-mining PubMed (https://pubmed.ncbi.nlm.nih.gov/) titles and abstracts and OMIM XML gene entries. We searched PubMed using the keywords “dominant negative” for the DN mechanism, “gain of function” OR “activating mutation” for the GOF mechanism, and “haploinsufficiency” OR “haploinsufficient” OR “dosage sensitivity” OR “dosage sensitive” OR “heterozygous loss of function” for the LOF mechanism. The same workflow was applied to OMIM entries. The resulting corpus was tokenized into sentences, and to facilitate downstream data curation, we filtered for lines that explicitly mention the keywords, thus keeping the most descriptive lines for each gene. The LOF class was appended with genes annotated in the ClinGen database (https://clinicalgenome.org/) as “Sufficient evidence for dosage pathogenicity” (as of 7 July 2022), and a supporting evidence was added from the ClinGen entry. The raw evidence lines were manually reviewed, and obvious false positives were removed. For example, in the LOF class, a line may be: “… individuals harboring a heterozygous deletion in ATAD3A are unaffected suggesting a dominant-negative pathogenic mechanism or a gain-of-function mechanism for de novo missense variants rather than haploinsufficiency” (*76*), which explicitly dismisses haploinsufficiency as a molecular mechanism. We noticed that a large proportion of GOF and DN evidence lines pertained to artificial constructs used in biological research and were not linked to human disease, requiring additional manual curation for verification. In overlap cases, when genes belong to multiple categories, we used a hierarchical strategy to create a nonredundant gene list: DN > GOF > LOF. We made available the genes of different molecular mechanism classes (table S1), containing also the evidence lines and the relevant PubMed identifiers.

### Protein functional classes

Functional classification of proteins was retrieved from PANTHER version 17.0 (http://www.pantherdb.org/). In the category “Transporter,” we included the classes “transporter,” “transmembrane signal receptor,” and “membrane traffic protein.” In the category “Metabolic enzymes,” we grouped “nucleic acid metabolism protein” and “metabolite interconversion enzyme.” Finally, the category “TF/chromatin regulator” represents the combined classes of “gene-specific transcriptional regulator” and “chromatin/chromatin-binding, or -regulatory protein.”

### Protein abundance

Protein abundances were obtained from the integrated human dataset (version 2021) of PAXdb (https://pax-db.org/). Parts per million (ppm) values were converted to molar concentration based on the equation given by (*77*).

### HEK293 active ribosome profile

Normalized active ribosome protected fragments in the Human Embryonal Kidney 293 lineage were determined by (*20*). The data are available via the National Center for Biotechnology Information (NCBI) Gene Expression Omnibus (https://www.ncbi.nlm.nih.gov/geo/) under accession GSE112353. Values were averaged over the two biological replicates, and transcripts with values <1 were excluded from the analysis.

### Cotranslationally assembling proteins in HEK293 cells

The gene set was downloaded from the supplemental material of (*3*).

### Coiled-coil motif–containing proteins

Coiled-coil motif–containing proteins were retrieved from UniProt, using the following search terms: (keyword:KW-0175) AND (organism_id:9606) AND (reviewed:true).

### Position of genes on chromosomes

Genes were mapped to chromosomes using the consensus coding sequence (CCDS) database (*78*) downloaded via the NCBI FTP site.

### Molecular graphics

Visualization of structures was performed with UCSF ChimeraX version 1.5 (*79*).

### Statistical analyses

Data exploration and statistical analyses were carried out in RStudio “Elsbeth Geranium” release, using R version 4.2.2. The R packages used for analyses were as follows: tidyverse, tidytable, rsample, rstatix, scales, ggridges, and ggbeeswarm. Error bars in bar charts represent 68% Jeffrey’s binomial confidence intervals, and the probabilities between the proportions of cotranslationally assembling subunits were calculated from the hypergeometric distribution. The 95% confidence interval for the OR was calculated with the SE method, where the value of the 97.5th percentile point of the normal distribution (~1.96) was derived as stats::qnorm(0.975) in R. In Wilcoxon rank sum tests, the effect size was defined as the *z* score computed from the *P* value over the square root of sample size. In multiple comparisons, the Holm-Bonferroni method was used to correct for familywise error rate. In the bootstrap analysis, data were stratified for molecular mechanisms in 10,000 resamples. The *P* value was calculated by determining the fraction of point estimates indicating a C-terminal interface shift, with correction for finite sampling. The 95% confidence intervals of the bootstrap estimates were derived using the percentile method.

#### Lasso regression—Feature selection

To prioritize genes that mainly give rise to non-LOF mutations over those that harbor LOF mutations, the following variables were included in the model:

1) gnomAD mutational constraint metrics (*40*):

• pLI—Probability that transcript falls into the distribution of haploinsufficient genes.

• pRec—Probability that transcript falls into distribution of recessive genes.

• oe_lof—Observed over expected ratio for predicted LOF variants in transcript.

2) Sequence-derived or evolutionary variables:

• Protein-length—As per UniProt canonical isoform.

• Number of paralogs (*39*)—Paralogs of human genes were called from Ensembl via the biomaRt R package.

• Maximum identity to paralog (*39*)—We calculated the protein sequence identity of each gene to every one of its paralogs using Clustal Omega version 1.2.4, and the maximum identity was kept.

• Human-macaque (*Macaca mulatta*) dN/dS (*39*)—The ratios of nonsynonymous to nonsynonymous substitutions between human-macaque orthologs were called from Ensembl via the biomaRt R package.

• ncGERP++—The phylogenetic conservation values of the genes’ regulatory sequences as derived from GERP++ scores were acquired from (*80*).

• Number of domains (*39*)—The number of domains was taken from the human proteome–specific Pfam release 15 November 2021.

3) Membrane propensity—We calculated from protein sequences the mean value of the scale NAKH900110 “Normalized composition of membrane proteins” from the AA index database (https://www.genome.jp/aaindex/).

4) Interaction network–based property:

• Betweenness centrality—This measure was calculated from the human protein interaction network of the STRING database version 11.5 at the default score threshold of 400 using the STRINGdb and igraph R packages.

5) Structural properties:

• Structural symmetry (monomer, heteromer, and homomeric symmetry groups: *C*_2_, *C*_*n*>2_, *D*_*n*>1_, and other homomeric symmetry), interface size, and number of subunits.

• Structure isoelectric point—as previously described in (*17*).

• Absolute contact order—We calculated the contact order from the AlphaFold predicted human structures using the perl script written by Eric Alm available at https://depts.washington.edu/bakerpg/contact_order/contactOrder.pl.

A copy of the script can be found in the OSF repository linked to this manuscript.

• Mean structure pLDDT and α-helix content.

6) Functional properties:

• Protein functional classification from this study. Proteins with functions other than those introduced earlier were classified as “known other function,” and those lacking a functional annotation were classed as “unknown function.”

7) Experimental data:

• Protein abundance.

• Cotranslational assembly annotations.

8) RNA expression variance—We accessed the rna_tissue_consensus.tsv.zip file from the Human Protein Atlas (https://www.proteinatlas.org/) on 1 September 2022 and calculated the variance in expression per gene across the 54 tissues.

#### Lasso regression—Data preparation

We assembled a dataset of 9051 genes with the above features. Genes of monomers in the PDB were assigned an interface size of 0, a relative interface location of 0, and a number of subunits of 1 and were assumed not to undergo cotranslational assembly even if they were detected by (*3*). Missing data in 10 variables were imputed using five nearest neighbors. The missing value rates were the following (percent missing in brackets): ncGERP++ (9.7); human-macaque dN/dS (9.3); pLI, pRec, and oe_lof (7.3); betweenness centrality (5.6); protein abundance (4.2); number of domains (2.7); structure isoelectric point (2.1); RNA expression variance (1.3). Last, all nominal variables were one-hot encoded and numeric data were normalized to have an SD of one and a mean of zero.

#### Lasso regression—Model building and performance evaluation

Logistic regression with lasso (least absolute shrinkage and selection operator) is a solution to fitting a model in which only certain variables play a role. The algorithm applies increasingly larger penalties to multivariable regression coefficients, shrinking those of less important variables to zero, causing their sequential dropout (L1 regularization) and thus retaining only informative features. First, to avoid inflating the model’s performance by the presence of homologs, we performed redundancy filtering at 50% sequence identity on the full canonical protein sequences using the method described in the “Relative interface location” section. This procedure removed 88 from the 879 genes with experimentally available structure data and dominant molecular mechanism classifications. The remaining 791 genes, of which 543 (69%) are non-LOF and 248 (31%) are LOF, were split into 75% training and 25% test sets with 10-fold cross-validation performed on the training set and repeated three times. The model was tuned using 18 values of the λ parameter, generated to be log-linearly distributed between 0 and 1. The final value of λ = 0.00501 was chosen on the basis that it yielded the highest prediction accuracy in the assessment folds of the cross-validation. The model was finalized on the entire training set and evaluated on the test set. The similarity of ROC areas under the curve (AUCs) measured on the cross-validation folds (0.735) versus on the test set (0.743) suggested that the model had not been overfitted. Variable importance was computed as the absolute values of the β coefficients scaled to the [0,1] interval. Model building and evaluation was performed using the tidymodels R metapackage. Thresholds T1 and T2 were derived using the threshold_perf() function from the R package probably.

## Notes

View/request a protocol for this paper from *Bio-protocol*.
