# Supplementary material for: Buffering of genetic dominance by allele-specific protein complex assembly
Source: Sci Adv. 2023 May 31;9(22):eadf9845. doi: 10.1126/sciadv.adf9845 (PMC10413657; doi:10.1126/sciadv.adf9845)
Supplement: Supplementary file 1 — Supplementary Text Figs. S1 to S4 Legends for tables S1 and S2 [file sciadv.adf9845_sm.pdf]

Science Advances
AAAS

Supplementary Materials for

Buffering of genetic dominance by allele-specific protein complex assembly

Mihaly Badonyi and Joseph A Marsh

Corresponding author: Joseph A Marsh, joseph.marsh@ed.ac.uk

Sci. Adv. **9**, eadf9845 (2023)
DOI: 10.1126/sciadv.adf9845

The PDF file includes:

Supplementary Text
Figs. S1 to S4
Legends for tables S1 and S2

Other Supplementary Material for this manuscript includes the following:

Tables S1 and S2

Supplementary Text

Additional controls for potential confounding variables in inheritance analyses

Protein length. Similar to the analysis of protein abundance in **Fig. 2B**, we controlled for protein length, because AD homomers and repeated subunits tend to be longer than AR (fig. S1D, $p = 7.1 \times 10^{-10}$; Wilcoxon rank-sum test). Since longer proteins take more time to translate and could form larger subunit interfaces than shorter proteins, the difference in length between AD and AR subunits would actually favour the cotranslational assembly of AD subunits. When we split the subunits into length quartiles, we found that AD subunits have a significantly lower proportion of cotranslational assembly across the four bins (fig. S1E), suggesting that the overall trend is not confounded by protein length.

Confidence in cotranslational assembly. Bertolini *et al.* have provided a confidence-based classification for the cotranslationally assembling proteins (*3*). However, relying entirely on the high confidence candidates is prohibitive to these analyses, as they make up only one-fifth of the detected proteins. To address potential biases coming from low confidence candidates, we divided the subunits into high and low confidence groups (fig. S1F). The high confidence group excludes all low confidence proteins, and similarly, the low confidence group excludes high confidence proteins altogether. The results showed a significant reduction in the level of cotranslational assembly in AD compared to AR subunits in both the high confidence group (OR = 0.66, $p = 7.7 \times 10^{-3}$) and the low confidence group (OR = 0.55, $p = 5 \times 10^{-10}$). One possible explanation for the stronger trend in the low confidence group is that they are enriched in membrane-bound complexes. These complexes tend to adopt cyclic symmetry, which has the strongest buffering capacity among the main symmetry groups, as demonstrated in **Fig. 2C**. On the other hand, high confidence candidates are limited to exclusively cytoplasmic or nuclear proteins by design (*3*), and thus may not reflect the full diversity of protein complexes.

Alpha-helix content. Coiled-coil motifs are highly enriched among cotranslationally assembling proteins (*3*). Our analysis showed that homomers and repeated subunits participating in cotranslational assembly are significantly enriched in alpha helices (effect size = 0.161, $p = 1.5 \times 10^{-52}$; Wilcoxon rank-sum test), and this remains unchanged even after the removal of coiled-coil motif containing proteins from the data (effect size = 0.155, $p = 9.6 \times 10^{-44}$). To account for a potential bias from alpha helix content, we divided the subunits into four quartiles and re-examined the trend (fig. S1G). The results support the trend across the bins, although with a lack of statistical significance in the quartile with the lowest alpha helix content.

Supplementary Figures

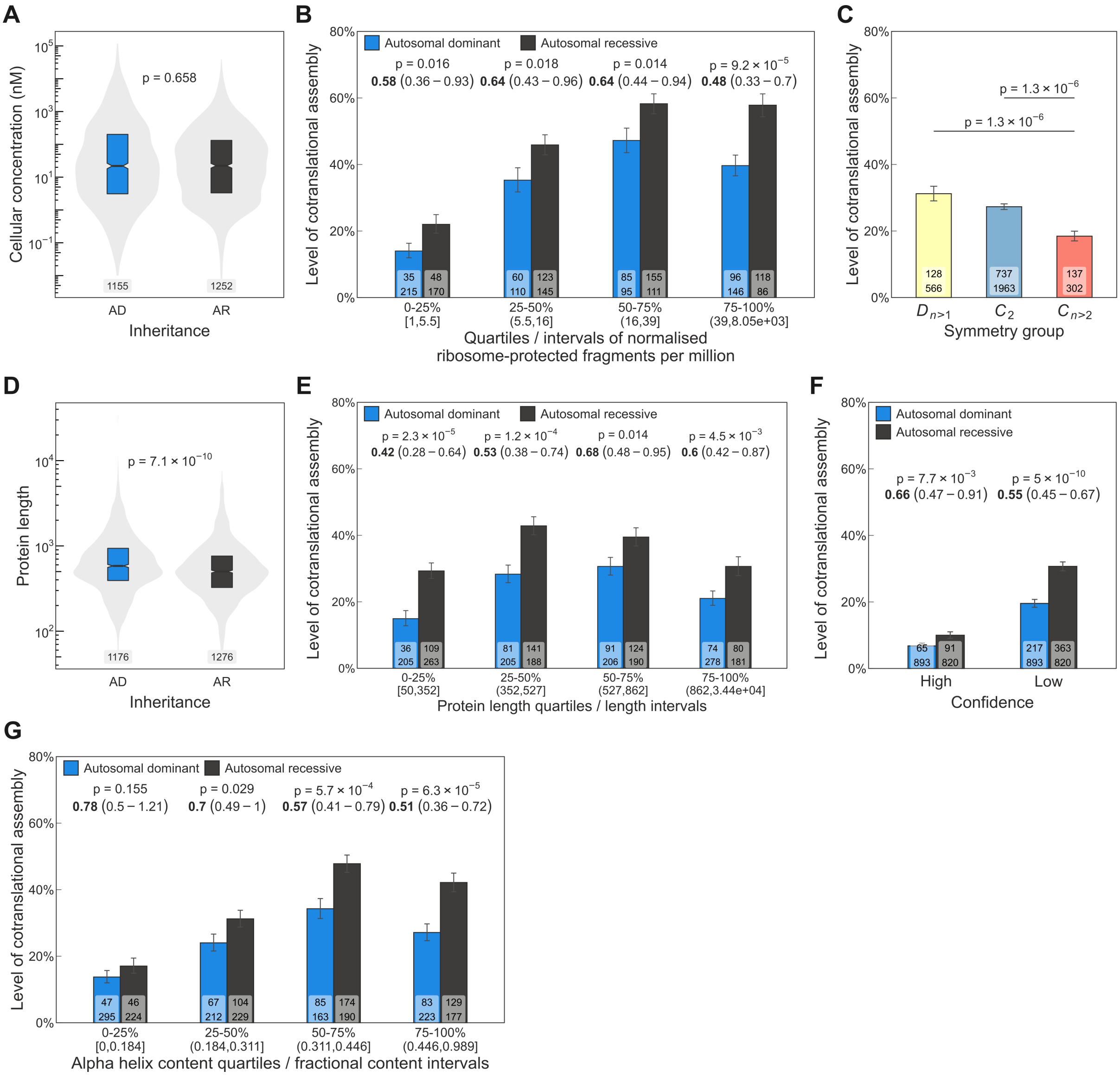

Fig. S1. Controls of potential confounders of the inheritance-level analysis.

(**A**) Box-violin plot comparison of the abundance distribution of AD and AR homomers and repeated subunits. Boxes denote data within 25th and 75th percentiles, the middle line represents the median and the notch contains the 95% confidence interval of the median. Numbers are sample size and the p-value was calculated with the Wilcoxon rank-sum test.

(**B**) The level of cotranslational assembly among AD vs AR genes binned into quartiles of active ribosome protected fragment counts measured in HEK293 cells. Each bin corresponds to 25% of proteins by count and the fragment per million intervals are displayed in brackets. Bar values are per cent level of cotranslational assembly, error bars are Jeffrey's 68% binomial credible intervals. The p-value from the hypergeometric test and the odds ratio (in bold) and its 95% confidence interval is shown above the bars. Labels on bars are the count of cotranslationally assembling subunits (top) and all other subunits (bottom). Panels **E-G** have the same parameters.

(**C**) Level of cotranslational assembly in homomeric symmetry groups.

(**D**) Box-violin plot comparison of the length distribution of AD and AR subunits. Numbers at the bottom represent sample size and the p-value was calculated with the Wilcoxon rank-sum test.

(**E**) The level of cotranslational assembly binned by protein length.

(**F**) The level of cotranslational assembly grouped by the confidence in their identification.

(**G**) The level of cotranslational assembly binned by fractional helix content.

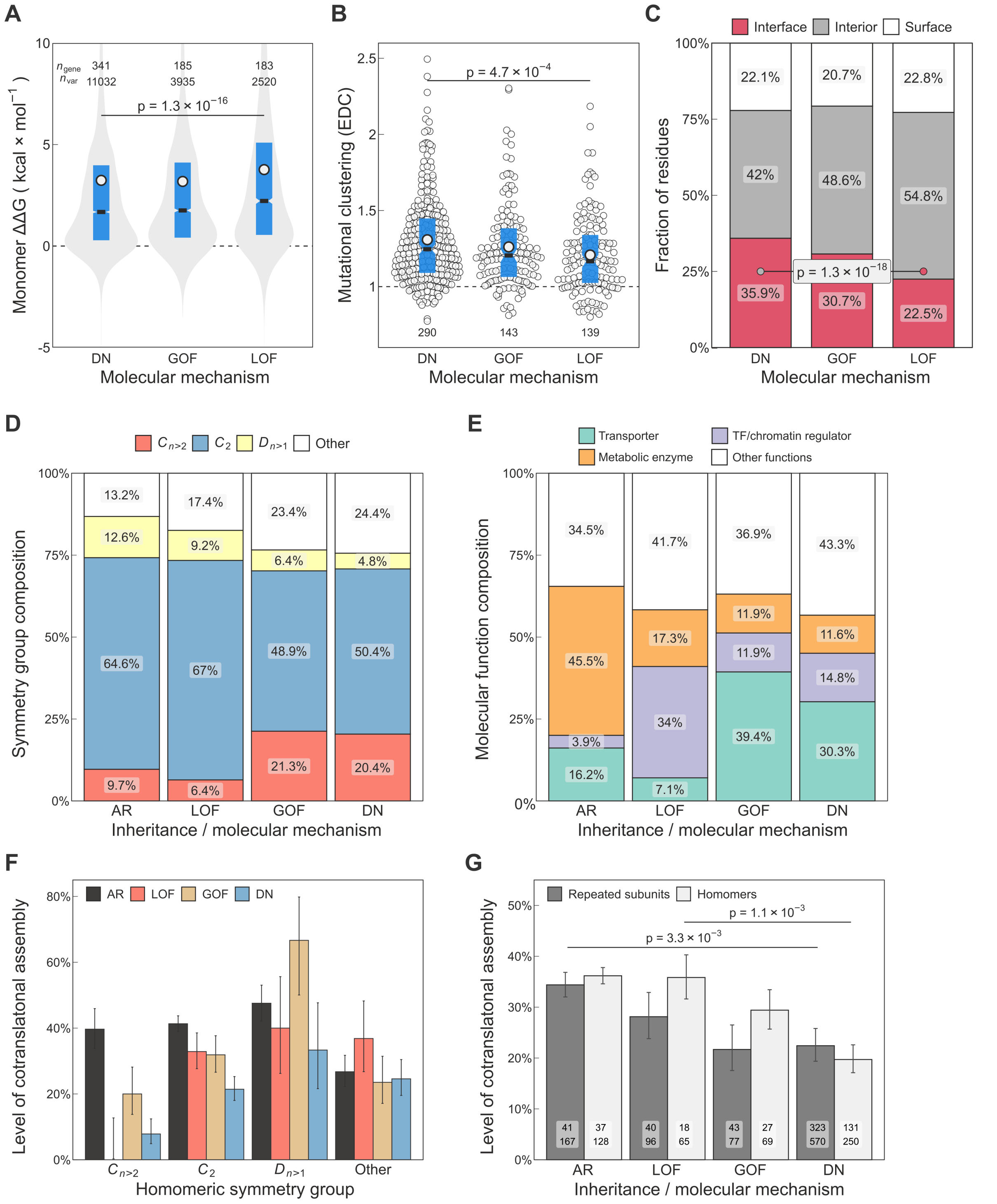

Fig. S2. Controls of potential confounders of the molecular mechanism-level analysis.

(**A**) Box-violin plot comparison of the predicted ΔΔG of pathogenic mutations in homomers and repeated subunits, grouped by molecular mechanisms. Boxes denote data within 25th and 75th percentiles, the middle line represents the median, the notch contains the 95% confidence interval of the median and white dots are the mean. Numbers on top are sample sizes for genes (n_{gene}) and missense variants (n_{var}) within the groups. The p-value was calculated with the Wilcoxon rank-sum test.

(**B**) Box-beeswarm plot comparison of the extent of disease clustering (EDC) metric that measures the extent to which pathogenic mutations cluster in 3D space. Numbers show the number of genes in each group. The p-value

was calculated with the Wilcoxon rank-sum test.

(**C**) Stacked bar chart showing the interface residue enrichment of missense pathogenic mutations in the DN group relative to LOF. The p-value was calculated with the hypergeometric test.

(**D**) Stacked bar chart of the symmetry group composition of homomeric subunits with different inheritance and molecular mechanisms.

(**E**) Stacked bar chart of the molecular function composition of homomers and repeated subunits with different inheritance and molecular mechanisms.

(**F**) Level of cotranslational assembly within the different inheritance and molecular mechanism classes subset by homomeric symmetry groups. Bar values are per cent level of cotranslational assembly, error bars are Jeffrey's 68% binomial credible intervals.

(**G**) Level of cotranslational assembly split into homomers and repeated subunit heteromers. Bar values are per cent level of cotranslational assembly, error bars are Jeffrey's 68% binomial credible intervals. The p-values were calculated from a hypergeometric test.

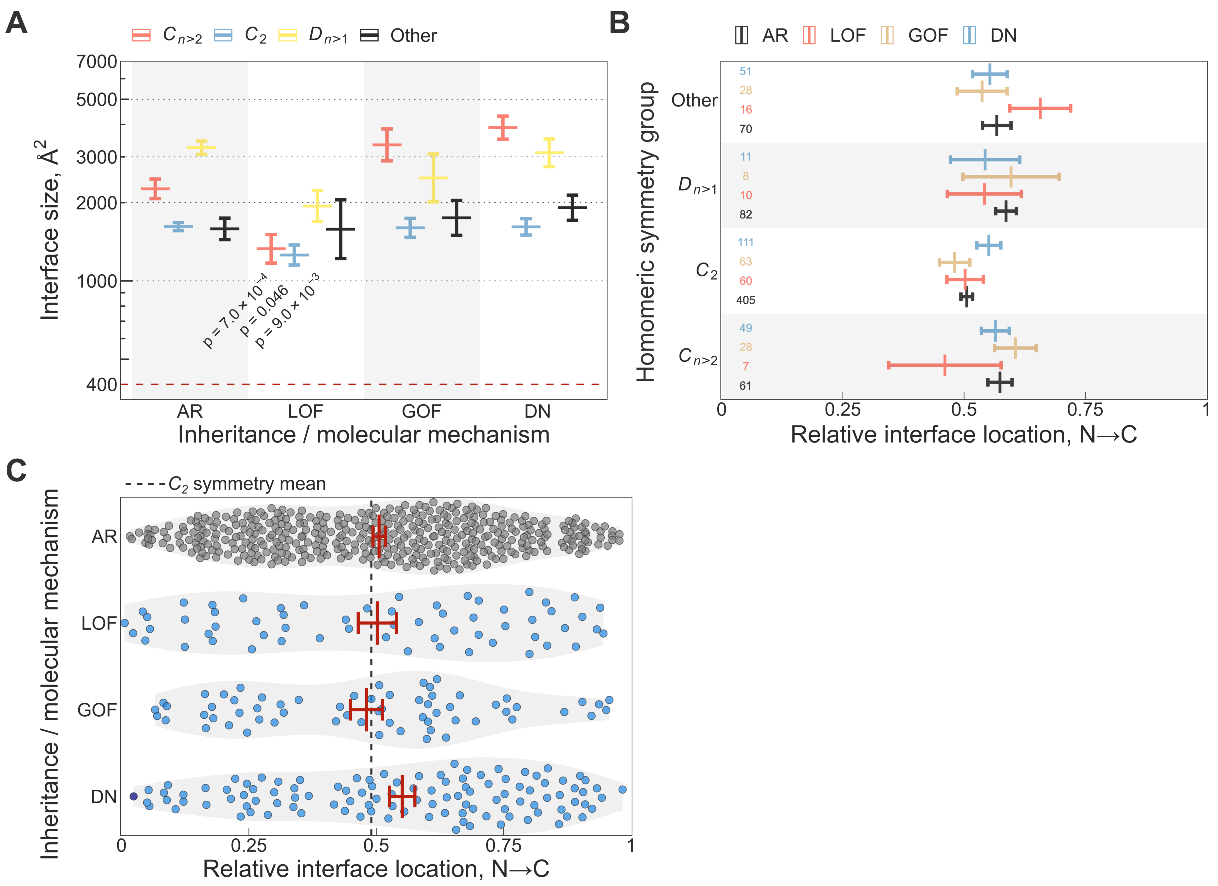

Fig. S3. Supplemental analyses using interface size and relative interface location.

(**A**) Interface area differences in homomers with different inheritance and molecular mechanisms. Crossbars are mean ± SEM. The p-values were calculated with Dunn's test using Holm-Bonferroni correction. Sample sizes are shown in panel **B**.

(**B**) Relative interface location of homomers with different inheritance and molecular mechanisms. Crossbars are mean ± SEM. Sample sizes are shown on the left.

(**C**) Relative interface location of C_2 symmetric homodimers with different inheritance and molecular mechanisms. Violins show the density distribution of the data and the crossbars are mean ± SEM. Dashed line shows the symmetry mean measured in all human C_2 dimers with structural data.

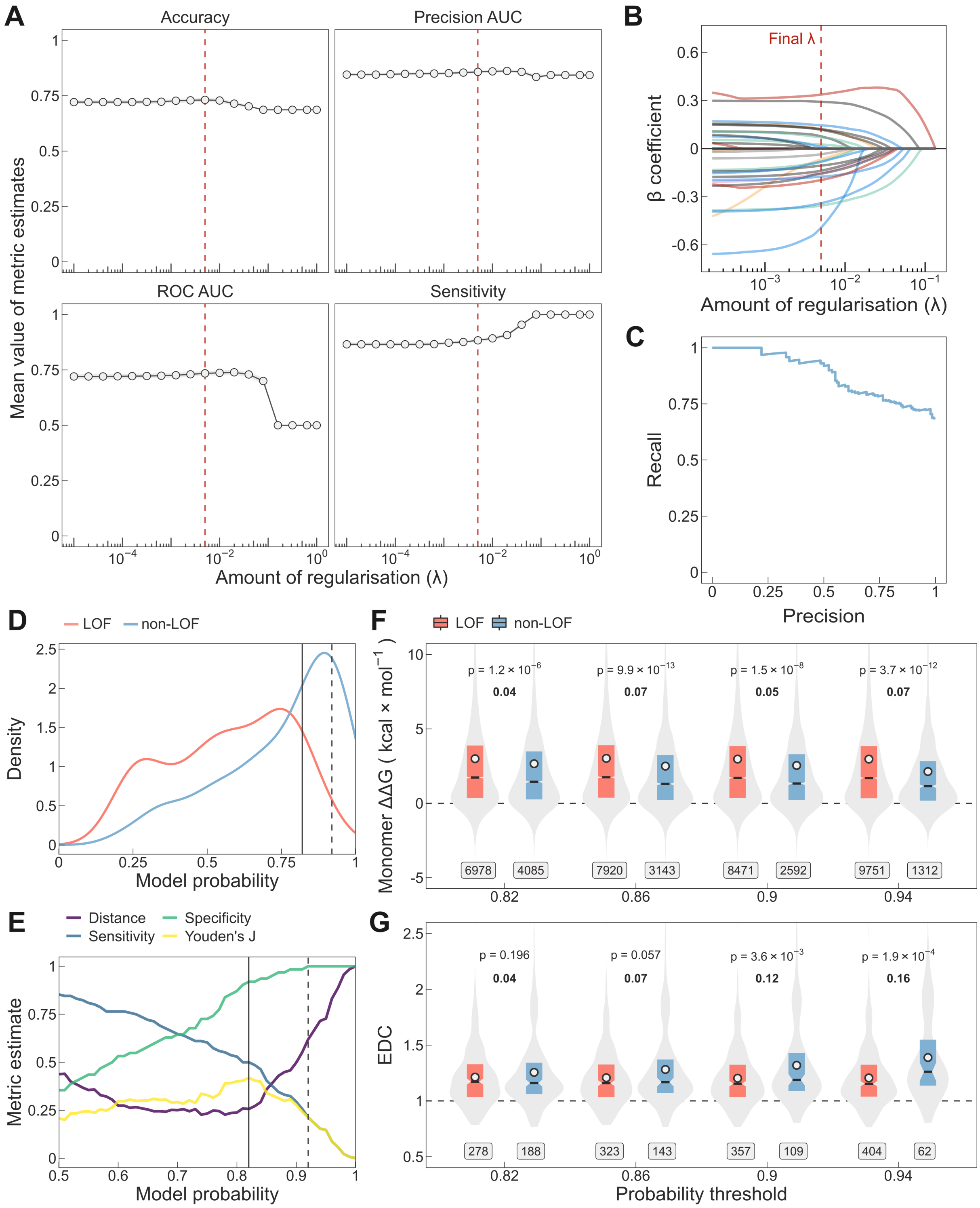

Fig. S4. Performance evaluation of the lasso regression model.

(**A**) Performance of the lasso regression model as a function of the penalty parameter estimated from the cross-validation folds. Dashed line is at λ = 0.00501, the penalty value in the final model.

(**B**) Lasso penalty (λ) vs the regression coefficient (β). The lines are coloured according to the type of the variable: sequence-derived or evolutionary variables (blue), functional annotations (green), mutational constraint metrics (red), structural properties (black), interaction network-based property (pink), and experimental data (orange).

(**C**) Precision-recall curve of the lasso regression model measured on the test set.

(**D**) Distribution of model probabilities for non-LOF and LOF genes in the test set. The solid vertical line marks the recommended threshold p = 0.82 (T1), the probability at which Youden's J statistic has its maximum value. The dashed line marks p = 0.92 (T2), the probability at which the model reaches maximum specificity.

(**E**) Performance metrics measured on the test set as a function of the model probability threshold. The distance is defined as (1 - sensitivity) ^ 2 + (1 - specificity) ^ 2 and Youden's J statistic as sensitivity + specificity - 1. Solid and dashed vertical lines are defined in (**D**).

(**F**) Differences in Gibbs free energy change and the extent of disease clustering (EDC) (**G**) of pathogenic mutations between proteins predicted to be non-LOF versus all other proteins measured at different thresholds. Genes that were used for training the model as well as known autosomal recessive genes were excluded. Boxes denote data within 25th and 75th percentiles with the middle line representing the median, the notch containing the 95% confidence interval of the median and the white dots are the mean. Labels indicate the number of variants ($\Delta\Delta G$) or the number of proteins (EDC) in the groups. The p-values were calculated with Wilcoxon rank-sum tests and effect sizes are shown in bold.

Supplementary Table Legends

Table S1. Autosomal dominant genes classified into protein-level dominant molecular disease mechanisms. The columns in the CSV file are as follows:

1. **gene**: HGNC gene symbol
2. **class**: molecular mechanism with levels *dn* (dominant negative), *gof* (gain of function) and *lof* (loss of function)
3. **pmid**: PubMed ID
4. **evidence_line**: supporting evidence line extracted from the article/OMIM/ClinGen

Table S2. Output of the lasso regression model. The columns in the CSV file are as follows:

1. **rank**: rank of the gene (by p_non_lof)
2. **gene**: HGNC gene symbol
3. **p_non_lof**: probability of the gene being associated with non-LOF mechanisms
4. **is_training**: whether the gene was part of the training set (1=yes, 0=no)
5. **is_AD**: whether the gene is associated with autosomal dominant disease inheritance (1=yes, 0=no)
6. **is_AR**: whether the gene is exclusively associated with autosomal recessive inheritance (1=yes, 0=no)
